# S-Klotho level and physiological markers of cardiometabolic risk in healthy adult men

**DOI:** 10.18632/aging.203861

**Published:** 2022-01-30

**Authors:** Agnieszka Żelaźniewicz, Judyta Nowak-Kornicka, Bogusław Pawłowski

**Affiliations:** 1 Department of Human Biology, University of Wrocław, Wrocław, Poland

**Keywords:** aging, healthspan, longevity, early marker, cardiometabolic risk

## Abstract

S-Klotho is perceived as a biomarker of healthy aging that has been shown to be inversely associated with cardiometabolic risk in elderly individuals. The aim of this study was to test if s-Klotho level is associated with cardiometabolic risk markers in younger healthy men in order to verify the possible role of s-Klotho level as an early marker of cardiometabolic risk. A cross-sectional study was conducted among 186 healthy men (M_age_=35.33, SD_age_=3.47) from a Western urban population. Serum basal levels of s-Klotho, lipid profile, homocysteine, glycemia markers, C-reactive protein, liver transaminases and creatinine were evaluated. Also, blood pressure was measured and cardiometabolic risk score and homeostatic model assessment for insulin resistance (HOMA-IR) were calculated. Testosterone and cortisol levels, self-reported psychological stress, physical activity, smoking in the past, alcohol use and body adiposity were controlled for. We found no relationship between levels of s-Klotho and physiological markers of cardiometabolic risk in the studied population. The results were similar when controlled for adiposity, testosterone level, physical activity, alcohol use and smoking in the past. We suggest that s-Klotho level is not an early marker of cardiometabolic risk in younger middle-aged healthy men.

## INTRODUCTION

The α-Klotho gene has been described as an “aging suppressor” gene in mice, encoding a protein, s-Klotho, with multiple pleiotropic effects [1]. A defect in mouse α-Klotho gene expression conferred shortened lifespan and progeroid phenotype linked with multiple disorders resembling human aging. This phenotype included organ and skin atrophy, infertility, atherosclerosis, vascular endothelial dysfunctions, vascular calcification, cardiac hypertrophy, decreased bone mineral density, sarcopenia, glucose metabolism disorders, and impaired cognition. In contrast, overexpression of α-Klotho gene significantly extended both healthspan and lifespan in mice [2–4].

The systemic effects of α-Klotho appear to be primarily exerted by its circulating form, soluble Klotho protein (s-Klotho), expressed predominantly in kidneys and to a lesser extent in the brain, skeletal muscle, urinary bladder, aorta, gonads, and thyroid gland [1]. S-Klotho functions as a humoral factor through binding to its cell-surface receptor and as an enzyme acting on cell-surface glycoproteins activating (e.g. calcium channel proteins) or inhibiting (e.g. phosphate transporters) cell surface receptors and enzymes in multiple tissues and organs [5–8]. In humans, s-Klotho has been shown to improve kidney functioning [9], systemic phosphate homeostasis [10] and glucose metabolism [11], to inhibit insulin/IGF-1 signaling pathways [12], to act as co-receptor of the fibroblast growth factor 23 [13], to decrease intracellular oxidative stress [14] and chronic inflammation [15–18]. Thus, the effect of s-Klotho on longevity and healthspan may result from its metabolic functions as hyperphosphatemia, oxidative stress, chronic inflammation, imbalanced glucose metabolism, decreased IGF-1 levels accelerate aging in humans [17–21]. As such, s-Klotho level has emerged as a biomarker of healthy ageing, inversely linked with chronological age and a number of age-related illnesses both in model organisms and in humans and an intriguing target for interventional studies [15, 22]. This is especially important as life expectancy has significantly increased in developed countries during the last century with the consequent increment of chronic disease incidence [23].

Oxidative stress and chronic inflammation are positively linked with such age-related conditions as type 2 diabetes and cardiovascular disorders [24, 25], the dominant metabolic diseases worldwide with expected increases in prevalence [23]. Due to its anti-inflammatory and anti-oxidant properties s-Klotho is expected to play a protective role against cardiometabolic diseases [26, 27] for instance by maintaining endothelial wall homeostasis and integrity, ameliorating vascular calcification, improving kidney functions [28–30] or insulin sensitivity [11]. However, the possible role of s-Klotho as an early marker of cardiometabolic risk has not been proven yet.

Previous research has shown that polymorphisms in the human Klotho gene are associated with various cardiovascular events and metabolic syndrome as well as with cardiometabolic risk factors, such as reduced HDL level and elevated blood pressure [28, 31]. Also, s-Klotho level has been shown to be inversely associated with the prevalence of cardiometabolic diseases in adults aged 24-102 years [26] or stress-induced cardiac hypertrophy and remodeling [32]. On the other hand, Liang et al. [33] found no relationship between s-Klotho level and hypertension or arterial stiffness in general Chinese population. One of the possible reasons for these contradictory results may be a differential effect of age on the association between s-Klotho and cardiometabolic disease [34]. Recently, Amaro-Gahete et al. [35] showed that s-Klotho level is negatively related with cardiovascular risk and insulin resistance markers level in a group of healthy men and women of 40-65 years but not in young adults of 18-25 years. This suggests that s-Klotho may serve as a marker of cardiometabolic status in middle-aged but not very young individuals. S-Klotho level has been also shown to be related with early indicators of atherosclerosis such as carotid artery intima-media thickness, flow-mediated dilation of brachial artery and epicardial fat thickness in healthy adults of median age 32 years, however on a relatively small sample size (N=50) [36]. S-Klotho level has been also shown to be inversely related with the probability of developing heightened glucose level and hyperglycemia in 80 males of 40-80 years [37]. Thus, so far s-Klotho level has been shown to be lower in individuals with cardiometabolic disease [26, 32] or to be related with cardiometabolic risk in individuals of older age [35], but to our knowledge no study has investigated the relationship between s-Klotho level and physiological markers of cardiometabolic risk in younger middle-aged men. If s-Klotho level is a reliable marker of current and future health in relatively young individuals it may help to identify individuals with an increased risk of developing age-related disorders and may serve as a measure of relative fitness, predicting disability in later life. Studies on early markers of age-related disorders are especially important for early interventions in western populations, where life expectancy has increased in recent decades, however, the fundamental aging process remains unchanged [38].

In this study we tested for the relationship between s-Klotho level and markers of cardiometabolic risk in healthy men between 30-45 years in order to verify if s-Klotho may be used as an early marker of cardiometabolic risk in this age range. Thus, we aimed to replicate the work of Amaro-Gahete et al. [35] on a group of young middle-aged men. Studies on anti-ageing factors in older middle-aged or elderly populations are compromised by the fact that the majority of participants already have some form of age-related disease, which may impact the levels of these factors [39]. Participants’ age range in this study (30-45 years) will allow to verify if the relationship between s-Klotho and cardiometabolic risk markers levels can also be detected in younger but not too young individuals. The age of onset of age-related cardiometabolic diseases decreases in western countries [40, 41], increasing in thirties [42, 43]. The onset of these disorders may occur even 9-12 years before its clinical diagnosis [44]. Thus, it is important to identify early markers of such diseases (and of the risk of their development) for prophylactic purposes, as they may no longer be diseases primarily of the elderly.

Cardiometabolic risk was evaluated with biomarkers for risk of developing cardiovascular problems, impaired glucose tolerance and liver dysfunction. As s-Klotho level decreases in individuals with kidney damage [45] we have controlled for possible renal dysfunctions based on estimated glomerular filtration rate (eGFR) and creatinine levels. We have also controlled for factors that have been shown to be related with s-Klotho level and cardiometabolic risk, such as chronological age [46], body composition [47], physical activity [48], testosterone level [49–51], alcohol use [52] and psychological stress level [53].

## RESULTS

### Descriptive statistics

Descriptive statistics of the main variables are presented in Table 1 (see also Supplementary Materials for descriptive statistics of additional variables – Supplementary Table 1).

**Table 1:** Descriptive statistics of the main variables (N=186).

	**M**	**SD**	**Min**	**Max**
Age [years]	35.33	3.47	29.73	44.29
S-Klotho [pg/ml]	1144.38	461.85	306.70	2686.50
BMI^1^ [kg/m2]	25.66	3.52	18.62	37.46
Cardiometabolic risk score	0.01	0.69	-1.47	2.90
Total cholesterol [mg/dl]	193.10	36.53	116.00	340.00
Homocysteine [μmol/l]	13.62	2.80	8.80	26.35
hsCRP^2^ [mg/L]	1.20	1.23	0.01	6.49
HbA1c^3^ [mmol/mol]	34.08	2.85	27.00	42.00
Glucose to insulin ratio	12.64	5.89	3.67	34.54
HOMA-IR^4^	2.12	1.19	0.41	7.04
AST/ALT	1.01	0.33	0.32	2.44
Creatinine [mg/dl]	0.94	0.11	0.65	1.40
Total testosterone [ng/dl]	487.15	174.86	135.30	1157.00
Stress [2–14]	7.66	2.34	2.00	14.00

^1^BMI, Body Mass Index.

^2^hsCRP, high sensitivity C-reactive protein level.

^3^HbA1c, glycated hemoglobin level.

^4^HOMA-IR, homeostatic model assessment for insulin resistance.

^5^Aspartate aminotransferase (AST) to alanine transaminase (ALT) ratio.

Individuals who smoked in the past had higher cardiometabolic risk score (M=0.25, SD=0.79) compared with individuals who have never smoked (M=-0.05, SD=0.66) (t(184)=-2.34, *p*=0.02). Also, individuals who smoked in the past had higher total cholesterol level (M=2.31, SD=0.07) compared with individuals who have never smoked (M=2.27, SD=0.08) (t(184)=-2.35, *p*=0.02). The two groups of men did not differ in terms of s-Klotho, mean blood pressure (MBP), triglycerides, high-density lipoprotein (HDL), homocysteine, hsCRP, glycated hemoglobin (HbA1c), glucose to insulin ratio, homeostatic model assessment for insulin resistance (HOMA-IR), creatinine, aspartate aminotransferase to alanine transaminase ratio (ALT/AST) or total testosterone level (in each case: p>0.09 – See Supplementary Table 2 in Supplementary Material).

Individuals who were physically active had lower cardiometabolic risk score (M=-0.12, SD=0.63) compared with individuals who were inactive (M=0.15, SD=0.74) (t(184)=-2.68, p=0.008). Individuals who were physically active had lower total cholesterol level (LOG values: M=2.26, SD=0.08) compared with individuals who were inactive (LOG values: M=2.30, SD=0.08) (t(184)=-4.11, p<0.001). Also, individuals who were physically active had lower high sensitivity C-reactive protein (hsCRP) level (LOG values: M=-0.26, SD=0.56) compared with individuals who were physically inactive (LOG values: M=-0.08, SD=0.54) (t(184)=-2.26, p=0.02). Individuals who were physically active had higher glucose to insulin ratio (LOG values: M=1.09, SD=0.21) and lower HOMA-IR level (LOG values: M=0.22, SD=0.24) compared with individuals who were inactive (LOG G:I: M=1.02, SD=0.19; LOG HOMA-IR: M=0.32, SD=0.22) (LOG G:I: t(184)=2.56, p=0.01; LOG HOMA-IR: t(184)=-2.90, p=0.004). Individuals who were physically active had higher ALT/AST ratio (M=0.01, SD=0.14) compared with individuals who were inactive (M=-0.05, SD=0.14) (t(184)=3.08, p=0.002). Individuals who were physically active had also higher total testosterone level (M=2.69, SD=0.14) compared with those who were inactive (M=2.63, SD=0.17) (t(184)=2.64, p=0.009). The two groups also differed in terms of triglycerides level, fat mass index, waist circumference, glucose, insulin and ALT levels but did not differ in terms of s-Klotho level, age, body mass index (BMI), lean mass, MBP, HDL, homocysteine, HbA1c, AST levels or stress (in each case: p>0.06 – See Supplementary Table 3 in Supplementary Materials).

Men who rarely drink alcohol had higher levels of s-Klotho level compared with men who sometimes and men who often drink alcohol (F(2,183)=6.90, *p*=0.001). Men who sometimes drink alcohol and men who often drink alcohol did not differ in terms of s-Klotho level (*p*>0.05). The three groups of men also differed in terms of triglycerides level but did not differ in terms of other measures of cardiometabolic risk (*p*>0.08 in each case – See Supplementary Table 4 in Supplementary Materials for detailed statistics).

S-Klotho level was not related with chronological age, BMI, fat or lean body mass, psychological stress, testosterone level, or creatinine level (Table 2). The results of correlation analysis for the relationship between age, testosterone, stress, BMI and cardiometabolic risk markers are presented in Supplementary Materials (Supplementary Table 5).

**Table 2:** The results of correlation analyses for the relationship between s-Klotho and markers of cardiometabolic risk and controlled variables (N=186).

	**r**	***p***
Age [years]	0.09	0.20
BMI^1^ [kg/m^2^]	0.02	0.78
Cardiometabolic risk score	-0.03	0.71
Total cholesterol LOG [mg/dl]	-0.05	0.47
Homocysteine LOG [μmol/l]	0.05	0.52
hsCRP^2^ LOG [mg/L]	-0.12	0.10
HbA1c^3^ [mmol/mol]	-0.02	0.78
Glucose to insulin ratio LOG	-0.06	0.41
HOMA-IR^4^ LOG	0.09	0.24
ALT/AST^5^ LOG	0.13	0.07
Creatinine LOG [mg/dl]	0.13	0.08
Total testosterone LOG [ng/dl]	0.14	0.06
Stress [2–14]	0.06	0.40

^1^BMI, Body Mass Index.

^2^hsCRP, high sensitivity C-reactive protein level.

^3^HbA1c, glycated hemoglobin level.

^4^HOMA-IR, homeostatic model assessment for insulin resistance.

^5^Aspartate aminotransferase (AST) to alanine transaminase (ALT) ratio.

### Klotho level and cardiovascular risk markers

There was no relationship between s-Klotho level and cardiometabolic risk score, total cholesterol, homocysteine or hsCRP level (Table 2). There was also no correlation between s-Klotho level and the level of clinical markers of glycemia or liver functioning (Table 2). None of the cardiometabolic risk markers was related with s-Klotho level. The results of correlation analysis between s-Klotho level and additional variables are presented in Supplementary Materials (Supplementary Table 6).

### Klotho level and cardiovascular risk markers, controlled for confounders

There was no relationship between s-Klotho level and cardiometabolic risk score (Table 3 - Model 1), total cholesterol (Table 3 - Model 2), homocysteine (Table 3 - Model 3), hsCRP (Table 3 - Model 4), HbA1c (Table 3 - Model 5), glucose to insulin ratio (Table 3 - Model 6), HOMA-IR (Table 3 - Model 7), when adjusted for age, BMI, alcohol use, testosterone level and physical activity. Also, s-Klotho was not related with MBP, triglycerides, HDL, glucose, insulin, ALT or AST (See Supplementary Materials for the detailed results - Supplementary Table 6). S-Klotho was positively related with ALT/AST ratio when adjusted for age, BMI, alcohol use, testosterone level and physical activity (Table 3 - Model 8), however, the p value would exceed the level of 0.05 after Bonferroni correction for multiple comparisons. The models using fat mass index or lean mass index instead of BMI showed similar results, thus are not presented.

**Table 3:** The results of regression analysis of the relationship between s-Klotho level and measures of cardiometabolic risk, adjusted for age, BMI, alcohol use, physical activity and testosterone level (N=186).

	**β**	**SE(β)**	**t(179)**	***p***
**Model 1: Dependent variable: cardiometabolic risk score: F(6,179)=41.65, adj. R^2^=0.57, *p*<0.001**				
S-Klotho LOG [pg/ml]	0.01	0.05	0.16	0.87
Age [years]	-0.003	0.05	-0.06	0.95
**BMI^1^ [kg/m^2^]**	**0.62**	**0.06**	**11.28**	**<0.001**
Alcohol use	0.05	0.05	1.08	0.28
Physical activity	-0.09	0.05	-1.79	0.07
**Total testosterone LOG [ng/dl]**	**-0.21**	**0.05**	**-3.79**	**<0.001**
**Model 2: Dependent variable: total cholesterol LOG: F(6,179)=9.77, adj. R^2^=0.22, *p*<0.001**				
S-Klotho LOG [pg/ml]	-0.02	0.07	-0.29	0.77
Age [years]	0.10	0.07	1.58	0.12
**BMI^1^ [kg/m^2^]**	**0.31**	**0.07**	**4,.22**	**<0.001**
Alcohol use	0.10	0.07	1.50	0.14
**Physical activity**	**-0.24**	**0.07**	**-3.60**	**<0.001**
Total testosterone LOG [ng/dl]	-0.08	0.07	-1.04	0.30
**Model 3 Dependent variable: Homocysteine LOG: F(6,179)=0.80, adj. R^2^<0.001, *p=0.57***				
S-Klotho LOG [pg/ml]	0.05	0.08	0.63	0.53
Age [years]	0.01	0.07	0.19	0.85
BMI^1^ [kg/m^2^]	0.15	0.08	1.80	0.07
Alcohol use	0.06	0.08	0.73	0.47
Physical activity	0.01	0.07	0.13	0.90
Total testosterone LOG [ng/dl]	0.06	0.08	0.67	0.50
**Model 4: Dependent variable: hsCRP LOG: F(6,179)=7.62, adj. R^2^=0.18, *p*<0.001**				
S-Klotho LOG [pg/ml]	-1.22	0.07	-1.73	0.08
Age [years]	-0.01	0.07	-0.10	0.92
**BMI^1^ [kg/m^2^]**	**0.43**	**0.08**	**5.62**	**<0.001**
Alcohol use	0.03	0.07	0.48	0.63
Physical activity	-0.12	0.07	-1.84	0.07
Total testosterone LOG [ng/dl]	0.07	0.08	0.86	0.39
**Model 5: Dependent variable:** HbA1c**: F(6,179)=1.99, adj. R^2^=0.03, *p*=0.07**				
S-Klotho LOG [pg/ml]	-0.04	0.08	-0.59	0.56
Age [years]	0.14	0.07	1.89	0.06
BMI^1^ [kg/m^2^]	0.01	0.08	0.07	0.94
Alcohol use	-0.13	0.08	-1.75	0.08
Physical activity	-0.07	0.07	-0.91	0.36
Total testosterone LOG [ng/dl]	-0.14	0.08	-1.71	0.09
**Model 6: Dependent variable:** Glucose to insulin ratio LOG**: F(6,179)=18.24, adj. R^2^=0.36, *p<*0.001**				
S-Klotho LOG [pg/ml]	-0.09	0.06	-1.42	0.16
Age [years]	0.02	0.06	0.44	0.66
**BMI^1^ [kg/m^2^]**	**-0.44**	**0.07**	**-6.59**	**<0.001**
Alcohol use	0.01	0.06	0.23	0.82
Physical activity	0.10	0.06	1.60	0.11
**Total testosterone LOG** [ng/dl]	**0.24**	**0.07**	**3.63**	**<0.001**
**Model 7: Dependent variable:** HOMA-IR LOG**: F(6,179)=19.99, adj. R^2^=0.38, *p*<0.001**				
S-Klotho LOG [pg/ml]	0.10	0.06	1.72	0.09
Age [years]	-0.01	0.06	-0.14	0.89
**BMI^1^ [kg/m^2^]**	**0.47**	**0.07**	**7.08**	**<0.001**
Alcohol use	-0.03	0.06	-0.56	0.57
**Physical activity**	**-0.12**	**0.06**	**-2.06**	**0.04**
**Total testosterone LOG** [ng/dl]	**-0.22**	**0.07**	**-3.34**	**0.001**
**Model 8: Dependent variable:** ALT/AST LOG**: F(6,179)=16.30, adj. R^2^=0.33, *p*<0.001**				
**S-Klotho LOG [pg/ml]**	**0.13**	**0.06**	**1.98**	**0.04**
Age [years]	0.09	0.06	1.45	0.15
**BMI^1^ [kg/m^2^]**	**-0.46**	**0.07**	**-6.70**	**<0.001**
Alcohol use	0.07	0.06	1.19	0.24
**Physical activity**	**0.14**	**0.06**	**2.27**	**0.02**
**Total testosterone LOG** [ng/dl]	**0.16**	**0.07**	**2.26**	**0.02**

^1^BMI, Body Mass Index.

Additionally, we have run a regression analysis to test for the relationship between s-Klotho and testosterone level. S-Klotho and testosterone level correlated positively when controlled for BMI, physical activity, alcohol use and age (Table 4 and Supplementary Table 7).

**Table 4:** The results of regression analysis of the relationship between s-Klotho and testosterone level, adjusted for age, BMI, alcohol use, and physical activity (F(5,180)=4.35*, adj. R^2^*=0.08, *p*<0.001, N=186).

	**β**	**SE(β)**	**t(180)**	***P***
Age [years]	0.10	0.07	1.36	0.18
BMI^1^ [kg/m^2^]	0.11	0.08	1.39	0.17
**Alcohol use**	**-0.27**	**0.07**	**-3.84**	**<0.001**
**Total testosterone LOG** [ng/dl]	**0.17**	**0.08**	**2.13**	**0.03**
Physical activity	0.03	0.07	0.47	0.64

^1^BMI, Body Mass Index.

## DISCUSSION

The results of our study showed no relationship between s-Klotho level and physiological markers of cardiometabolic risk in healthy, non-smoking men form an urban, western population, aged 30-45 years. The results were similar when controlled for BMI, testosterone level, physical activity, alcohol use and smoking in the past.

Previous research has shown that s-Klotho may be an important marker of ongoing cardiovascular disease [26, 31, 54, 55] - although see also [33] and [56] for no relationship] or even a therapeutic agent for the treatment of CVD [57]. However, the results of our and previous studies suggest that its role as an early marker of cardiovascular disease risk in healthy individuals may depend on age. Amaro-Gahete et al. [35] showed a negative relationship between s-Klotho and cardiometabolic risk markers levels in middle-aged adults of 40-65 years but not in young adults of 18-25 years. Semba et al. [26, 54] reported a strong inverse association between S-Klotho level and the likelihood of developing cardiovascular disease, as well as risk of all-cause mortality, in a large cohort of adults aged over 65 years. We suggest that s-Klotho level may serve as an early marker of cardiometabolic risk in healthy middle-aged adults (at least over 40 years) but not in relatively young adults. Further large-scale prospective studies are required to verify the relationship between s-Klotho and cardiometabolic risk markers levels in different age groups of healthy individuals.

We found also no relationship between s-Klotho level and lifestyle-related risk factors of cardiometabolic disease such as smoking in the past, lack of physical activity or self-assessed psychological stress. On the other hand, these factors (except psychological stress) were related with cardiometabolic risk score, lipid profile and in case of physical activity also with markers of glucose metabolism, liver functioning and hsCRP level. This may suggest that these markers may be more reliable risk factors of cardiometabolic risk in healthy adult men between 30 and 45 years compared with s-Klotho level. S-Klotho level was only negatively related with frequency of alcohol drinking, confirming the results of previous studies [52].

The positive relationship between s-Klotho and testosterone level was observed when adjusted for age, BMI, physical activity, and alcohol use. Furthermore, testosterone level was negatively related with majority of cardiometabolic risk biomarkers level (except homocysteine level). Ageing beyond 35-40 years is associated with gradual decline in testosterone level in men [58] and low testosterone level appears to be a biomarker of poor health in older men associated with acute and chronic pathologies, including cardiometabolic disorders, and also with high risk of all-cause mortality [49, 50, 59, 60]. Previous research showed that testosterone increases Klotho gene expression and increased Klotho expression up-regulates the nuclear androgen receptor in mice [61]. Dote-Montero et al. [51] also showed a positive correlation between s-Klotho and testosterone level in healthy middle-aged sedentary adults (45-65 years old), suggesting that testosterone may up-regulate α-Klotho gene expression via androgen receptor. Here we show that this relationship can also be observed in younger men (30-45 years old).

Although we did not find a significant relationship between s-Klotho and cardiometabolic risk markers levels it cannot be excluded that genetic polymorphisms of the klotho gene may be associated with these factors also in relatively young, healthy individuals, what would require further studies. Previous research showed that single nucleotide polymorphisms in the klotho gene are correlated with s-Klotho levels and the susceptibility to hypertension or coronary artery disease [2, 62, 63], cancer risk and longevity [64]. The results on a relationship between Klotho polymorphism and glucose homeostasis are not consistent [65, 66]. What is interesting, the relationship between Klotho polymorphism and aggravation of cardiometabolic disease is enhanced with age [34] what might confirm earlier presumption that s-Klotho level may be related with cardiometabolic risk only in middle-aged or older adults but not in relatively young individuals.

Interpreting these results requires an understanding of the limitations of the study. First, because of the cross-sectional design of the study no firm conclusions of causal association can be drawn from this study. Second, s-Klotho and cardiometabolic risk markers levels were assayed only once. Despite the rigorous study protocol we cannot exclude the possibility of some intra-individual variability in the measured markers, especially as little is known about intra-individual variation of s-Klotho (e.g. daily fluctuations, etc.). The design of the future studies should include repeated measurements of s-Klotho and cardiometabolic risk markers levels. Finally, it is possible that some discrepancies in the results of the studies on the relationship between s-Klotho level and other measures of health may be due to the differences in methods used for s-Klotho measurement [67]. Hiejboer et al. [67] evaluated the quality of the three commercially available s-Klotho assays and showed poor inter-assay as well as intra-assay agreement between them. IBL assay (used in this study) was the only one that provided information on the epitopes against which their antibodies are directed, offering the best results after evaluation of different tests including within-run variation, between-run variation, matrix effects, linearity, and recovery. However, as s-Klotho is relatively novel marker these differences should be factored in when comparing the results of various studies.

In conclusion, our study showed no relationship between s-Klotho and cardiometabolic risk markers level in healthy, non-smoking adults between 30 and 45 years. Nonetheless, future prospective studies should investigate the importance of s-Klotho measurements in cardiovascular risk stratification and its possible role as an early marker of the risk of developing cardiometabolic health.

## MATERIALS AND METHODS

This cross-sectional study is part of a larger research project focused on men’s health, conducted on 209 men (M_age_=35.26, SD_age_=3.49) from a western, urban population. Participants were recruited via information in local media or social networks. The study protocol and methodology were designed according to the Declaration of Helsinki (2013) and approved by local ethics committee. An informed written consent was obtained for participation in the study and use of data for scientific purposes from all participants.

### Participants

The criteria for inclusion for this study were as follows: age between 30-45 years, no diagnosed chronic disease (diabetes, cardiovascular disease, autoimmunological, etc.), no hormonal treatment, not smoking, no ongoing infections. Current health status was assessed based on blood morphology with smear and C-reactive protein (CRP) level. From the initial group of participants, 24 were excluded from the analyses due to the following reasons: a) age below 30 years (N=2); b) elevated CRP level (CRP > 10 μg/ml), indicating ongoing inflammatory state (N=1); c) missing data (N=20). Renal function was evaluated based on eGFR and creatinine levels. All participants had eGFR value above 60 ml/min/m2 (the lowest value was 63.40 ml/min/m2) and creatine within the clinical norm. Thus, the final analyses included 186 healthy men of age between 29.73 and 44.29 years (M_age_=35.33, SD_age_=3.47).

### Procedures

Fasting blood sample was taken between 7:30 a.m. and 9:00 a.m. for blood biochemical and hormone analyses. Participants were asked to refrain from physical activity, heavy meals and alcohol for 24 hours before the study visit. The participants completed questionnaire about demographic data, current and past health problems, medications, smoking and alcohol drinking pattern. Among the participants, 36 individuals declared that they had regularly smoked in the past (quitted at least one year prior the visit) and 150 stated that they had never smoked. Participants were also asked about the level of physical activity: the number and length (in minutes) of trainings per week and the type of sport practiced. The type of sport activities practiced by the participants were comparable in terms of intensity (running, biking, swimming, football, basketball, calisthenics, tennis, squash, CrossFit, strength workout). The participants were divided into two categories: 1) physically active (N=99), which included individuals who declared regular physical activity at least 60 mins/week; 2) and inactive (N=87), which included participants with no regular sport activity. There were no professional sportsmen in the study sample. Participants were also asked how often they drink alcohol and based on their answers they were divided into three groups: 1) rarely – i.e. once per month or less often (N=49), 2) sometimes - i.e. 2-4 times per month (N=81), 3) often – i.e. 2-3 times per week (N=56). Participants were also asked to estimate how stressful is their work and their personal life on scales from 1 (not stressful at all) to 7 (extremely stressful). The two scores were summarized resulting in self-perceived stress variable with the range from 2 to 14.

### Anthropometry

Height was measured twice with a classic stadiometer. The mean value of two measurements was used in the analysis. Weight was measured digitally (SECA mBCA 515) and BMI was calculated as kg/m^2^. Additionally, fat and lean body mass were measured with bioimpedance analysis (SECA mBCA 515). Fat and lean mass indexes were calculated as fat mass and lean body mass in kg divided by height in meters^2^. Waist circumference was measured twice at the midpoint between the lower border of the rib cage and the iliac crest (c.a. at the level of the umbilicus) with a flexible tape. The mean of the two measurements was used in the analysis.

### Klotho and hormone measurements

Blood samples were drawn after overnight fasting. After centrifugation serum was collected and stored at –80° C. Serum soluble circulating Klotho (s-Klotho) level was measured with enzyme-linked immunosorbent assay (ELISA) using commercial kits (IBL® Code no 27998; with inter-assay and intra-assay coefficient of variation less than 11.4% and less than 3,5% respectively with assay sensitivity of 6.15 pg/ml), following the manufacturer’s protocol. Calibrators (standards supplied with the kit) and serum samples were assayed in duplicate and the average absorbance value was used to calculate hormone concentration. Standard curve was created by plotting mean absorbance values for each standard (Y axis) against its concentration (X axis). Total s-Klotho concentration was calculated in relation to standard curve, multiplied by dilution ratio and expressed in pg/ml.

Quantitative measurement of total testosterone were assayed by certified analytical laboratory (DIAGNOSTYKA®) using Cobas analyzer and expressed in ng/dl.

### Cardiometabolic risk indices

Morning blood pressure was measured in a sitting resting position with a digital sphygmomanometer and standard protocol. Readings were taken twice and the mean of the two measurements was calculated. Mean blood pressure (MBP) was calculated from mean systolic (SBP) and mean diastolic (DBP) blood pressure according to the formula: MBP = 1/3 (SBP-DBP) + DBP.

Cardiometabolic risk score was calculated based on waist circumference, MBP, glucose, HDL-C and triglycerides levels, the clinical criteria for defining the risk of metabolic syndrome suggested by the International Diabetes Federation [68]. The values were standardized based on a formula: (value-mean)/standard deviation. To indicate greater cardiometabolic risk via increasing values, the standardized HDL-C values were multiplied by -1. Cardiometabolic risk scores were calculated as arithmetic mean of these 5 standardized values and lower values indicate a better cardiometabolic risk profile. Additionally, cardiometabolic risk was estimated based on total cholesterol [69], homocysteine [70], and hsCRP levels [71].

Glucose, HDL-C, triglycerides and total cholesterol levels were assayed in serum in a certified laboratory (DIAGNOSTYKA) using spectrophotometry and Cobas analyzer. Homocysteine level was assayed in a certified laboratory (DIAGNOSTYKA) using turbidimetric method with Cobas analyzer. hsCRP level was assayed in the laboratory at Department of Human Biology (UWr) using enzyme-linked immunosorbent assay and ELISA kit (DEMEDITEC® DE740011; inter- and intra-assay coefficient of variation were less than 6.3% and less than 6.9% respectively with assay sensitivity of 0.02μg/ml) in accordance to a user's manual. After series of incubation with conjugate solution and chromogen solution the reaction was stopped, and the absorbance value was read on ASYS UVM340 spectrophotometers with λ=450nm. The standard curve was plotted with the average absorbance value (y axis) of each standards against its concentration (x axis). The participant's hsCRP levels were calculated in relation to standard curve and expressed in μg/ml.

Insulin sensitivity was estimated based on glycated hemoglobin level [72], the homeostatic model assessment of insulin resistance index (HOMA-IR) [73], and fasting glucose to insulin level ratio. Fasting insulin level was measured in serum according to the procedure IB/LAB/100 in a certified laboratory (DIAGNOSTYKA). Glycated hemoglobin (HbA1c) level was assayed in blood with HPLC method with Variant analyzer.

Liver function, related with cardiometabolic risk, was evaluated based on AST/ALT ratio [74]. ALT and AST levels were assayed in a certified laboratory (DIAGNOSTYKA).

Creatinine level was measured to control for renal functions. Creatinine level was assayed in a certified laboratory (DIAGNOSTYKA).

### Statistical analyses

Based on the visual inspection of graphs, kurtosis and skewness values the distribution of levels of s-Klotho, triglycerides, total cholesterol, homocysteine, hsCRP, glucose, insulin, HOMA-IR, ALT, AST, creatinine, testosterone, glucose to insulin ratio, and ALT/AST ratio were assessed as non-normally distributed. Thus, these variables were log-transformed in order to obtain normal or near normal distribution.

T-test was used to verify if participants who smoked in the past (N=36) and participants who have never smoked (N=150) differed in terms of s-Klotho or cardiometabolic risk markers levels. T-test was also used to verify if participants who were classified as physically active (N=99) and participants who were classified as inactive (N=87) differed in terms of s-Klotho and cardiometabolic risk markers levels. ANOVA was used in order to verify if participants differed in terms of levels of s-Klotho and cardiometabolic risk markers levels depending on how often they drink alcohol: (1) rarely (N=49); 2) sometimes (N=81); 3) often (N=56). Tukey test was used as a post-hoc test. Pearson correlation was used in order to verify if s-Klotho level is related with controlled variables (chronological age, adiposity measures, testosterone and psychological stress level).

First, the relationship between s-Klotho and cardiometabolic risk markers levels was verified with Pearson correlation analysis. Then, a simple linear regression model was used to test the association between S-Klotho and cardiometabolic risk markers levels, adjusting for age, body composition, alcohol use, testosterone level and physical activity. Stress was not included as it was not related with any of the measures of cardiometabolic risk or s-Klotho level.

Analyses were performed with Statistica 12.0 software. The results were interpreted as statistically significant if *p*<0.05.

## Supplementary Material

Supplementary Tables
