# Supplementary material for: S-Klotho level and physiological markers of cardiometabolic risk in healthy adult men
Source: Aging (Albany NY). 2022 Jan 30;14(2):708–27. doi: 10.18632/aging.203861 (PMC8833136; doi:10.18632/aging.203861)
Supplement: Supplementary Tables [file aging-14-203861-s001.pdf]

SUPPLEMENTARY TABLES

Supplementary Table 1. Descriptive statistics of additional variables (N=186).

	M	SD	Min	Max
MBP[1] [mm Hg]	93.13	9.75	67.83	119.67
Triglycerides [mg/dl]	121.62	80.75	37.00	668.00
Lean mass index [kg/m^2]	19.61	1.64	14.95	24.44
Fat mass index [kg/m^2]	6.05	2.48	1.43	15.86
Waist circumference [cm]	92.00	10.29	69.50	129.00
HDL[2] [mg/dl]	53.62	11.97	29.00	88.00
Glucose [mg/dl]	9.13	4.68	2.20	26.30
Insulin [µIU/ml]	92.30	8.00	71.90	122.70
ALT[3] [U/l]	27.31	16.68	8.00	120.00
AST [U/l]	23.99	8.29	11.00	61.00

[1]MBP, Mean blood pressure.
[2]HDL, High density protein.
[3]ALT, Alanine transaminase.
[4]AST, Aspartate aminotransferase.

Supplementary Table 2. The difference in mean values of levels of s-Klotho, markers of cardiometabolic risk and controlled variables between men who had smoked in the past (N=36) and men who have never smoked (N=150).

	Never have smoked		Smoked in the past		t(184)	*p*
	M	SD	M	SD		
Age [years]	35.33	3.59	35.34	2.96	-0.03	0.98
BMI[1] [kg/m^2]	25.52	3.50	26.25	3.61	-1.12	0.26
**Waist circumference [cm]**	**91.21**	**10.33**	**95.27**	**9.59**	**-2.14**	**0.03**
Lean mass index [kg/m^2]	19.58	1.62	19.74	1.74	-0.53	0.60
Fat mass index [kg/m^2]	5.94	2.45	6.51	2.60	-1.25	0.21
s-Klotho LOG [pg/ml]	3.02	0.17	3.03	0.17	-0.43	0.66
MBP[1] [mm Hg]	92.60	9.61	95.35	10.15	-1.53	0.13
Triglycerides LOG [mg/dl]	2.01	0.22	2.08	0.24	-1.72	0.09
HDL[3] LOG [mg/dl]	1.72	0.09	1.72	0.10	0.01	0.99
Homocysteine LOG [µmol/l]	1.12	0.08	1.14	0.10	-0.85	0.40
hsCRP[4] LOG	-0.20	0.55	-0.08	0.56	-1.11	0.27
HbA1c[5] [mmol/mol]	33.91	2.87	34.81	2.68	-1.71	0.09
Glucose to insulin ratio LOG	1.06	0.20	1.04	0.19	0.54	0.59
**Glucose LOG [mg/dl]**	**1.96**	**0.03**	**1.98**	**0.04**	**-2.34**	**0.02**
Insulin LOG [µIU/ml]	0.90	0.22	0.94	0.21	-0.90	0.37
HOMA-IR[6] LOG	0.25	0.24	0.30	0.23	-1.19	0.24
ALT/AST[7] LOG	-0.01	0.14	-0.05	0.15	1.66	0.10
**ALT[8] LOG [U/l]**	**1.36**	**0.22**	**1.46**	**0.20**	**-2.59**	**0.01**
**AST[9] LOG [U/l]**	**1.35**	**0.13**	**1.41**	**0.13**	**-2.43**	**0.02**
Creatinine LOG [mg/dl]	-0.03	0.05	-0.04	0.05	1.46	0.15
Total testosterone LOG [ng/dl]	2.66	0.15	2.64	0.18	0.63	0.53
Stress [2-14]	7.65	2.20	7.72	2.89	-0.17	0.86

[1]BMI, Body mass index.
[2]MBP, Mean blood pressure.
[3]HDL, High density lipoprotein.
[4]hsCRP, High sensitivity C-reactive protein.
[5]HbA1c, Glycated hemoglobin.
[6]HOMA-IR, Homeostatic model assessment for insulin resistance.
[7]Aspartate aminotransferase (AST) to alanine transaminase (ALT) ratio.
[8]ALT, Alanine transaminase.
[9]AST, Aspartate aminotransferase.

Supplementary Table 3. The difference in mean values of levels of s-Klotho, markers of cardiometabolic risk and controlled variables between physically active (N=99) and non-active men (N=87).

	Physically active		Non-active		t(184)	*p*
	M	SD	M	SD		
Age [years]	35.22	3.75	35.45	3.15	-0.43	0.66
BMI [kg/m^2]	25.31	3.16	26.06	3.87	-1.46	0.15
**Waist circumference [cm]**	**90.29**	**9.56**	**93.94**	**10.80**	**-2.44**	**0.02**
Lean mass index [kg/m^2]	19.70	1.56	19.51	1.73	0.81	0.42
**Fat mass index [kg/m^2]**	**5.61**	**2.26**	**6.56**	**2.63**	**-2.64**	**0.009**
s-Klotho LOG [pg/ml]	3.03	0.18	3.01	0.15	0.68	0.50
**Cardiometabolic risk score**	**-0.12**	**0.63**	**0.15**	**0.74**	**-2.68**	**0.008**
MBP[1] [mm Hg]	92.88	9.98	93.42	9.53	-0.37	0.71
**Triglycerides LOG [mg/dl]**	**1.98**	**0.21**	**2.06**	**0.24**	**-2.40**	**0.02**
**Total cholesterol LOG [mg/dl]**	**2.26**	**0.08**	**2.30**	**0.08**	**-4.11**	**<0.001**
HDL[2] LOG [mg/dl]	1.73	0.09	1.70	0.10	1.89	0.06
Homocysteine LOG [μmol/l]	1.13	0.08	1.12	0.09	0.09	0.93
**hsCRP[3] LOG**	**-0.26**	**0.56**	**-0.08**	**0.54**	**-2.26**	**0.02**
HbA1c[4] [mmol/mol]	33.81	2.66	34.39	3.05	-1.39	0.16
**Glucose to insulin ratio LOG**	**1.09**	**0.21**	**1.02**	**0.19**	**2.56**	**0.01**
**Glucose LOG [mg/dl]**	**1.96**	**0.03**	**1.97**	**0.04**	**-2.26**	**0.02**
**Insulin LOG [μIU/ml]**	**0.87**	**0.22**	**0.95**	**0.20**	**-2.77**	**0.006**
**HOMA-IR[5] LOG**	**0.22**	**0.24**	**0.32**	**0.22**	**-2.90**	**0.004**
**ALT/AST[6] LOG**	**0.01**	**0.14**	**-0.05**	**0.14**	**3.08**	**0.002**
**ALT[7] LOG [U/l]**	**1.34**	**0.19**	**1.42**	**0.24**	**-2.42**	**0.02**
AST[8] LOG [U/l]	1.35	0.13	1.36	0.13	-0.66	0.51
Creatinine LOG [mg/dl]	-0.02	0.05	-0.04	0.05	1.86	0.06
**Total testosterone LOG [ng/dl]**	**2.69**	**0.14**	**2.63**	**0.17**	**2.64**	**0.009**
Stress [2-14]	7.64	2.26	7.69	2.46	-0.15	0.88

[1]MBP, Mean blood pressure.
[2]HDL, High density lipoprotein.
[3]hsCRP, High sensitivity C-reactive protein.
[4]HbA1c, Glycated hemoglobin.
[5]HOMA-IR, Homeostatic model assessment for insulin resistance.
[6]Aspartate aminotransferase (AST) to alanine transaminase (ALT) ratio.
[7]ALT, Alanine transaminase.
[8]AST, Aspartate aminotransferase.

Supplementary Table 4. The results of ANOVA for the difference in terms of the level of s-Klotho, cardiometabolic risk factors, and controlled variables between men who rarely drink alcohol (group 1: N=49), sometimes (group 2: N=81), and men who often drink alcohol (group 3: N=56).

	Model	G1(Never)	G2(Sometimes)	G3(Often)
Age [years]	F(2,183)=2.80, *p*=0.06	ns	ns	ns
BMI[3] [kg/m^2]	F(2,183)=0.78, *p*=0.46	ns	ns	ns
Waist circumference [cm]	F(2,183)=1.46, *p*=0.23	ns	ns	ns
Lean mass index [kg/m^2]	F(2,183)=0.21, *p*=0.81	ns	ns	ns
Fat mass index [kg/m^2]	F(2,183)=0.91, *p*=0.40	ns	ns	ns
**s-Klotho LOG [pg/ml]**	**F(2,183)=6.90, *p*=0.001[1]**	**3.09±0.17**	**3.02±0.16**	**2.97±0.16**
Cardiometabolic risk score	F(2,183)=1.41, *p*=0.25	ns	ns	ns
MBP[4] [mm Hg]	F(2,183)=1.88, *p*=0.16	ns	ns	ns
**Triglycerides LOG [mg/dl]**	**F(2,183)=4.21, *p*=0.02[2]**	**1.94±0.17**	**2.03±0.23**	**2.06±0.24**
Total cholesterol LOG [mg/dl]	F(2,183)=2.00, *p*=0.14	ns	ns	ns
HDL[5] LOG [mg/dl]	F(2,183)=0.24, *p*=0.78	ns	ns	ns
Homocysteine LOG [µmol/l]	F(2,183)=0.70, *p*=0.50	ns	ns	ns
hsCRP[6] LOG	F(2,183)=0.97, *p*=0.38	ns	ns	ns
HbA1c[7] [mmol/mol]	F(2,183)=1.44, *p*=0.24	ns	ns	ns
Glucose to insulin ratio LOG	F(2,183)=0.22, *p*=0.80	ns	ns	ns
Glucose LOG [mg/dl]	F(2,183)=0.40, *p*=0.67	ns	ns	ns
Insulin LOG [µIU/ml]	F(2,183)=0.22, *p*=0.80	ns	ns	ns
HOMA-IR[8] LOG	F(2,183)=0.22, *p*=0.80	ns	ns	ns
ALT/AST[9] LOG	F(2,183)=0.01, *p*=0.99	ns	ns	ns
ALT[10] LOG [U/l]	F(2,183)=0.91, *p*=0.40	ns	ns	ns
AST[11] LOG [U/l]	F(2,183)=2.53, *p*=0.08	ns	ns	ns
Creatinine LOG [mg/dl]	F(2,183)=1.37, *p*=0.26	ns	ns	ns
Total testosterone LOG [ng/dl]	F(2,183)=0.51, *p*=0.60	ns	ns	ns
Stress [2-14]	F(2,183)=0.82, *p*=0.44	ns	ns	ns

[1]The difference between G1 and G2 was significant (p=0.04); The difference between G1 and G3 was significant (p<0.001). The difference between G2 and G3 is not significant (p=0.25).
[2]The difference between G1 and G2 was not significant (p=0.06); The difference between G1 and G3 was significant (p=0.01). The difference between G2 and G3 was not significant (p=0.71).
[3]BMI, Body mass index.
[4]MBP, Mean blood pressure.
[5]HDL, High density lipoprotein.
[6]hsCRP, High sensitivity C-reactive protein.
[7]HbA1c, Glycated hemoglobin.
[8]HOMA-IR, Homeostatic model assessment for insulin resistance.
[9]Aspartate aminotransferase (AST) to alanine transaminase (ALT) ratio.
[10]ALT, Alanine transaminase.
[11]AST, Aspartate aminotransferase.

Supplementary Table 5. The relationship between age, testosterone, stress, BMI and cardiometabolic risk markers (N=186).

	Age		LOG tT		Stress		BMI	
	**r**	***p***	**r**	***p***	**r**	***p***	**r**	***p***
Age [years]			<0.01	0.98	0.01	0.88	**0.15**	**0.047**
BMI[1] [kg/m^2]	**0.15**	**0.047**	**-0.44**	**<0.001**	0.06	0.43		
Waist circumference [cm]	**0.18**	**0.01**	**-0.47**	**<0.001**	0.04	0.60	**0.89**	**<0.001**
Lean mass index [kg/m^2]	**0.23**	**0.001**	**-0.19**	**0.009**	0.06	0.41	**0.77**	**<0.001**
Fat mass index [kg/m^2]	0.05	0.46	**-0.50**	**<0.001**	0.04	0.57	**0.91**	**<0.001**
Cardiometabolic risk score	0.09	0.20	**-0.50**	**<0.001**	0.03	0.69	**0.73**	**<0.001**
MBP[2] [mm Hg]	0.02	0.81	**-0.17**	**0.02**	-0.04	0.56	**0.36**	**<0.001**
Triglycerides LOG [mg/dl]	0.06	0.43	**-0.54**	**<0.001**	0.06	0.45	**0.54**	**<0.001**
Total cholesterol LOG [mg/dl]	**0.16**	**0.03**	**-0.27**	**<0.001**	0.09	0.24	**0.40**	**<0.001**
HDL[3] LOG [mg/dl]	0.03	0.66	**0.39**	**<0.001**	-0.02	0.76	**-0.43**	**<0.001**
Homocysteine LOG [µmol/l]	0.04	0.54	<0.01	0.96	-0.08	0.29	0.13	0.07
hsCRP[4] LOG	0.05	0.49	**-0.17**	**0.02**	-0.01	0.85	**0.41**	**<0.001**
HbA1c[5] [mmol/mol]	0.013	0.08	**-0.16**	**0.03**	0.01	0.86	0.08	0.26
Glucose to insulin ratio LOG	-0.05	0.51	**0.45**	**<0.001**	<0.01	0.96	**-0.56**	**<0.001**
Glucose LOG [mg/dl]	0.10	0.19	**-0.18**	**0.01**	0.05	0.46	**0.32**	**<0.001**
Insulin LOG [µIU/ml]	0.06	0.41	**-0.45**	**<0.001**	0.01	0.86	**0.57**	**<0.001**
HOMA-IR[6] LOG	0.07	0.33	**-0.44**	**<0.001**	0.02	0.78	**0.58**	**<0.001**
ALT/AST[7] LOG	0.04	0.64	**0.40**	**<0.001**	<0.01	0.96	**-0.52**	**<0.001**
ALT[8] LOG [U/l]	0.04	0.57	**-0.36**	**<0.001**	-0.05	0.48	**0.55**	**<0.001**
AST[9] LOG [U/l]	0.11	0.15	**-0.15**	**0.04**	-0.08	0.26	**0.33**	**<0.001**
Creatinine LOG [mg/dl]	0.02	0.81	**0.15**	**0.04**	-0.04	0.56	-0.05	0.50
Testosterone LOG [ng/dl]	<0.01	0.98			-0.09	0.24	**-0.44**	**<0.001**
Stress [2-14]	0.01	0.88	-0.09	0.24			0.06	0.43

[1]BMI, Body mass index.
[2]MBP, Mean blood pressure.
[3]HDL, High density lipoprotein.
[4]hsCRP, High sensitivity C-reactive protein.
[5]HbA1c, Glycated hemoglobin.
[6]HOMA-IR, Homeostatic model assessment for insulin resistance.
[7]Aspartate aminotransferase (AST) to alanine transaminase (ALT) ratio.
[8]ALT, Alanine transaminase.
[9]AST, Aspartate aminotransferase.
Significant results are bolded.

Supplementary Table 6. The results of correlation analyses for the relationship between LOG s-Klotho and markers of cardiometabolic risk and controlled variables (N=186).

	**r**	***p***
Waist circumference [cm]	0.03	0.67
Triglycerides LOG	-0.14	0.06
Lean mass index [kg/m^2]	0.03	0.64
Fat mass index [kg/m^2]	0.01	0.93
MBP[1] [mm Hg]	0.01	0.93
HDL[2] LOG [mg/dl]	0.08	0.28
Glucose LOG [mg/dl]	0.11	0.13
Insulin LOG [µIU/ml]	0.07	0.31
ALT[3] LOG [U/l]	-0.04	0.62
AST[4] LOG [U/l]	0.08	0.25

[1]MBP, Mean blood pressure.
[2]HDL, High density lipoprotein.
[3]ALT, Alanine transaminase.
[4]AST, Aspartate aminotransferase.

Supplementary Table 7. The results of regression analysis for the relationship between s-Klotho and various measures of cardiometabolic risk, adjusted for age, BMI, alcohol use, physical activity and testosterone level (N=186).

	β	SE(β)	t(179)	*p*
**Model 1: Dependent variable: MBP: F(6,179)=5.07, adj. R^2=0.12, *p*<0.001**				
S-Klotho LOG [pg/ml]	0.03	0.07	0.48	0.63
Age [years]	-0.04	0.07	-0.64	0.52
**BMI[1] [kg/m²]**	**0.35**	**0.08**	**4.47**	**<0.001**
Alcohol use	0.12	0.07	1.61	0.11
Physical activity	0.01	0.07	0.12	0.90
Total testosterone LOG [ng/dl]	-0.01	0.08	-0.16	0.87
**Model 2: Dependent variable: triglycerides LOG: F(6,179)=22.95, adj. R^2=0.42, *p*<0.001**				
S-Klotho LOG [pg/ml]	-0.06	0.06	-1.01	0.32
Age [years]	-0.001	0.06	-0.01	0.99
**BMI[1] [kg/m²]**	**0.36**	**0.06**	**5.68**	**<0.001**
**Alcohol use**	**0.14**	**0.06**	**2.31**	**0.02**
Physical activity	-0.06	0.06	-1.13	0.26
**Total testosterone LOG [ng/dl]**	**-0.35**	**0.06**	**-5.48**	**<0.001**
**Model 3: Dependent variable: HDL LOG: F(6,179)=9.90, adj. R^2=0.22, *p*<0.001**				
S-Klotho LOG [pg/ml]	0.06	0.07	0.88	0.38
Age [years]	0.07	0.07	1.13	0.26
**BMI[1] [kg/m²]**	**-0.34**	**0.07**	**-4.67**	**<0.001**
Alcohol use	0.05	0.07	0.76	0.45
Physical activity	0.06	0.07	0.88	0.38
**Total testosterone LOG [ng/dl]**	**0.22**	**0.07**	**2.94**	**0.004**
**Model 5: Dependent variable: Glucose LOG: F(6,179)=4.90, adj. R^2=0.11, *p*<0.001**				
S-Klotho LOG [pg/ml]	0.10	0.07	1.30	0.19
Age [years]	0.05	0.07	0.64	0.52
**BMI[1] [kg/m²]**	**0.29**	**0.08**	**3.65**	**<0.001**
Alcohol use	-0.07	0.07	-0.95	0.34
Physical activity	-0.13	0.07	-1.81	0.07
Total testosterone LOG [ng/dl]	-0.04	0.08	-0.52	0.60
**Model 6: Dependent variable: Insulin LOG: F(6,179)=19.84, adj. R^2=0.38, *p*<0.001**				
S-Klotho LOG [pg/ml]	0.10	0.06	1.61	0.11
Age [years]	-0.02	0.06	-0.28	0.78
**BMI[1] [kg/m²]**	**0.46**	**0.07**	**6.98**	**<0.001**
Alcohol use	-0.02	0.06	-0.41	0.68
**Physical activity**	-0.11	0.06	-1.89	0.06
Total testosterone LOG [ng/dl]	**-0.23**	**0.07**	**-3.54**	**<0.001**
**Model 7: Dependent variable: ALT LOG: F(6,179)=14.51, adj. R^2=0.30, *p*<0.001**				
S-Klotho LOG [pg/ml]	-0.01	0.06	-0.15	0.88
Age [years]	-0.03	0.06	-0.55	0.59
**BMI[1] [kg/m²]**	**0.48**	**0.07**	**6.91**	**<0.001**
Alcohol use	0.05	0.06	0.73	0.47
Physical activity	-0.10	0.06	-1.63	0.10
Total testosterone LOG [ng/dl]	-0.12	0.07	-1.68	0.10
**Model 8: Dependent variable: AST LOG: F(6,179)=4.97, adj. R^2=0.11, *p*<0.001**				
S-Klotho LOG [pg/ml]	0.12	0.07	1.63	0.10
Age [years]	0.04	0.07	0.56	0.57
**BMI[1] [kg/m²]**	**0.30**	**0.08**	**3.77**	**<0.001**
**Alcohol use**	**0.16**	**0.07**	**2.17**	**0.03**
Physical activity	-0.02	0.07	-0.25	0.80
Total testosterone LOG [ng/dl]	-0.02	0.08	-0.31	0.75

[1]BMI, Body mass index.
